# Relationship between mental health and the quality of sleep during the first self-restraint in Japanese workers: a cross-sectional survey

**DOI:** 10.1080/21642850.2022.2112583

**Published:** 2022-08-16

**Authors:** Maki Furutani, Tianqi Guo, Kenji Hall, Xiongzhengjie Zhou

**Affiliations:** Graduate School of Human Development and Environment, Kobe University, Kobe-city, Japan

**Keywords:** Sleep, loneliness, anxiety, depression, mental health

## Abstract

**Objective:**

A few surveys have indicated that behavioural restrictions during the coronavirus disease 2019 pandemic have affected sleep and mental health. This study examined (1) the change in sleep-wake habits before and during the first self-restraint in Japan, (2) the factors that affect mental health, and (3) the model of mental health affecting the sleep quality, of workers.

**Design:**

A cross-sectional internet survey.

**Outcome Measures:**

A total of 512 Japanese workers self-assessed their sleep quality, loneliness, anxiety, and depression during self-restraint. Their previous sleep habits were also assessed.

**Results:**

Sleep habits remained almost regular, but 35.7% of participants reported poor sleep quality. Additionally, among the participants, 82.2% reported social loneliness, 37.9% reported emotional loneliness, 25.6% reported anxiety moods, and 49.0% reported depressive moods. Anxiety and depression were influenced by emotional and social loneliness, and marital status. These results showed that social and emotional loneliness worsened sleep quality with anxiety and depression. On the contrary, emotional loneliness had a significant and direct effect on sleep quality but not on social loneliness.

**Conclusion:**

This study shows that psychological factors affect subjective sleep quality during self-restraint. Psychological factors, such as loneliness, anxiety, and depression should be considered when maintaining good sleep quality under self-restraint.

## Introduction

Many countries have adopted measures of isolation and restriction of activities to prevent the spread of coronavirus disease 2019 (COVID-19) and these measures have changed people’s lifestyles. The new lifestyle requires people to refrain from shopping, entertainment, sports, dining with others, using public transport, attending events, and avoiding contact with other people as much as possible (WHO, 2021, p. 10, 1). In particular, teleworking, rotational work, staggered working hours, and online meetings have been encouraged. These lifestyle changes may have made it difficult to apply traditional stress coping strategies, and for workers, this might have led to more care and concern about unfamiliar communication methods. Consequently, people reported deteriorated mental health and sleep quality, including higher rates of anxiety or depressive symptoms, lower well-being, and increased loneliness (Cellini, Canale, Mioni, & Costa, 2020; González-Sanguino et al., 2020; Gualano, Lo Moro, Voglino, Bert, & Siliquini, 2020; Huang & Zhao, 2020; Li et al., 2020; Marelli et al., 2021; Mazza et al., 2020; Pai & Vella, 2021).

In Italy, the first European country to have a lockdown, 42.2% people reported sleep disturbance (17.4% reported moderate or severe insomnia), 23.2% people reported anxiety symptoms, and 24.7% reported depressive symptoms during the last two weeks of lockdown (Gualano et al., 2020). In addition, Gualano et al. (2020) indicated that being female facilitated sleep disturbance and anxiety symptoms, while avoidance of activity facilitated anxiety and depressive symptoms. On the other hand, being married and the absence of work-related troubles acted as inhibitors of anxiety, and older age was associated with less depression. Although the proportions of each symptom and their inhibitors or facilitators have been shown, there were no clear correlations between sleep, anxiety, and depression, including demographic variables.

Regarding the relationship between insomnia and mental disorders, previous studies have indicated that the chronicity of insomnia causes depression (Drake, Pillai, & Roth, 2014; Ohayon & Roth, 2003), suggesting that anxiety disorders appear in the same way as or after the onset of insomnia (Ohayon & Roth, 2003). Thus, the causal association between mental disorders and chronic insomnia is unclear. However, the first instance of self-restraint in Japan is an acute stressor for the nation. If insomnia, anxiety, and depression are observed simultaneously in Japan, as in Italy, it could be assumed that anxiety or depression symptoms cause insomnia, rather than assuming that chronic insomnia causes mental disorders.

In particular, the worker’s work environment vastly changed, possibly leading to worse mental health because undesirable social distancing increases loneliness and affects physical and mental health (Campagne, 2019). Unlike most developed countries that acted against COVID-19 in 2020, the Japanese government had few legal enforcement mechanisms to support its public health measures (Wright, 2021). However, in early 2020, the government released guidelines such as avoiding closed spaces, crowded places, and close-contact settings, and exercising self-restraint by avoiding going outside and working from home where possible, with which most Japanese people complied (Wright, 2021). GPS location information indicated an estimated 50% reduction in human mobility and 70% reduction in physical social contact in Tokyo in the week following the declaration of the first state of emergency in April 2020 (Yabe et al., 2020). The first declaration of state of emergency in Japan by the government (7 April 2020–25 May 2020) was a response to the Covid pandemic and requested self-restraint on the part of individuals by avoiding going outside. In Japan, although self-restraint was not mandatory, the suddenness of restrictions on socialising with others led to increased loneliness and caused anxiety or depression.

We conducted a web-based survey of the adult population in this study. The working-age population has a variety of background factors that affect mental health and sleep, such as employment status, marital status, and presence of children. We aimed to examine the model of mental health affecting the sleep quality of workers during their first self-restraint in Japan. First, we examined the changes in the workers’ sleep habits during and before self-restraint. Second, we detected the background factors associated with mental health during the behavioural restrictions. Furthermore, we identified a model by which mental health affects sleep quality. This study aimed to build a model of relationships rather than construct a theoretical model. In particular, we wanted to clarify whether there was a mediating effect on mental health.

## Materials and methods

### Design and participants

The study protocol was approved by the Human Ethics Committee of the Graduate School of Human Development and Environment at Kobe University, Japan (No. 436–2). The study protocol was approved by the Human Ethics Committee. We conducted a cross-sectional internet survey of 1,000 Japanese adults aged 20 years or above who had registered for the monitor market research, which had more than one million registered monitors. The participants were randomly selected within the allocation of a region of residence, gender, and age. The required sample size consisted of 384 workers and non-workers each, in order to be calculated at a confidence level of 95% with a standard error of 5.0 and an expected response rate of 50%. Of the 1,000 participants, 562 were workers (working full-time, working part-time, and self-employed/contractor), 393 were non-workers (students, homemakers, unemployed, retired, volunteers, etc.), and 45 were others. Although this study also surveyed non-workers, their lifestyles differed significantly from that of workers; the average time spent home on a weekday by Japanese people from before the COVID-19 pandemic was 12.5 h for workers, 19.9 h for housemakers, and 19.7 h for those aged 70 and over (NHK Broadcasting Culture Research Institute, 2006). Therefore, comparing behavioural changes between these groups due to the pandemic was considered impossible, and only workers were the focus of this study. All the respondents completed the DJGLS and HAD, but 50 respondents had incomplete answers for PSQI and sleep habits (during and before self-restraint). Thus, a total of 512 workers who completed all the surveys were finally analysed for the study.

Participants answered the questionnaires on the Internet from 18 May 2020 to 21 May 2020. This period was the last week of the first declaration of a state of emergency in Japan (7 April 2020–25 May 2020), which was a measure to prevent the COVID-19 pandemic. Under these conditions, all residents were required to (1) refrain from going out, except when necessary to maintain their lives (e.g. going to a hospital, buying groceries), (2) close schools (except nursery schools), and (3) restrict the use of places where people gather (e.g. department stores, movie theatres). However, Japan’s restrictions were milder, requiring only self-restraint, rather than lockdowns or other mandatory measures.

### Measures

The respondents were presented with an explanation of the purpose and objectives of the survey and information on privacy protection, and informed that participation was voluntary. Those who provided consent were directed to the survey page. Information that could identify individuals was not collected. To ensure that correct responses were provided for sleep habits and mental health during and before the self-restraint period, the questionnaire was divided into sections by period and included explanatory texts. The survey company checked for incorrect or inappropriate responses and logical inconsistencies, and then delivered the screened data. In addition, the authors checked and corrected any problems in the answers, such as sleep habits (e.g. 12-hour notation instead of 24-hour notation).

#### Demographic variables

Demographic variables were age, sex, education status, marital status, employment status, physical isolation experience, children’s age (0-5 years or 6–17 years), chronic disease, mental disorders, sleep disorder, and acute illness (Table 1). Except for age, the data were categorised as 1 for ‘yes’ and 0 for ‘no’. Age was used as a quantitative variable in all cases.

**Table 1.** Means, standard deviations, and frequencies of main study variables (*n* = 512).

	n	%			M	SD	Median	(Min – Max)
Age			Age		46.84	13.40	46.00	(20-83)
20<39 year	172	33.6	Before self-restraint					
40<59 year	239	46.7	Bed time	weekday	23:27	1:20	23:30	(18:30-30:00)
60 ≦ year	101	19.7	Rise time	weekday	6:28	1:11	6:30	(3:00-13:30)
Gender			Sleep duration	weekday	6:33	1:03	6:30	(3:15-9:30)
Male	317	61.9	Bed time	weekend	23:43	1:28	23:30	(18:30-30:30)
Female	195	38.1	Rise time	weekend	7:21	1:40	7:00	(3:00-14:00)
Educational level			Sleep duration	weekend	7:09	1:14	7:15	(1:00-12:15)
University education	285	55.7	During self-restraint					
Others	214	41.8	Bed time	weekday	23:25	1:25	23:00	(18:30-30:00)
Marital status			Rise time	weekday	6:33	1:14	6:30	(2:00-13:30)
Married	285	55.7	Sleep duration	weekday	6:41	1:04	6:45	(3:15-10:00)
Others	226	44.1	Bed time	weekend	23:45	1:35	23:38	(18:30-30:30)
Employment status			Rise time	weekend	7:30	1:44	7:15	(2:00-14:00)
Full-time	388	75.8	Sleep duration	weekend	7:15	1:10	7:15	(3:15-11:45)
Others	116	22.7	Sleep quality (PSQI global score)		4.95	2.50	5	(0-16)
Children aged 0–5 years			PSQI C1		1.09	0.65	1	(0-3)
Yes	42	8.2	PSQI C2		0.90	0.90	1	(0-3)
No	470	91.8	PSQI C3		1.34	0.85	1	(0-3)
Children aged 6–17 years			PSQI C4		0.14	0.47	0	(0-3)
Yes	91	17.8	PSQI C5		0.94	0.57	1	(0-3)
No	421	82.2	PSQI C6		0.12	0.54	0	(0-3)
Chronic disease			PSQI C7		0.41	0.65	0	(0-3)
Yes	437	12.2	Social loneliness (DJGLS)		2.40	1.03	3	(0-3)
No	61	87.8	Emotional loneliness (DJGLS)		1.21	1.05	1	(0-3)
Mental disorder			Anxiety (HAD)		5.56	3.34	5	(0-18)
Yes	46	9.2	Depression (HAD)		7.37	4.04	7	(0-19)
No	454	90.8						
Sleep disorder								
Yes	30	6.0						
No	469	94.0						
Acute illness								
Yes	8	1.6						
No	500	98.4						

n = number; M = mean; SD = standard deviation; PSQI C1 = sleep quality; PSQI C2 = sleep latency; PSQI C3 = sleep duration; PSQI C4 = habitual sleep efficiency; PSQI C5 = sleep disturbances; PSQI C6 = use of sleeping medication; PSQI C7 = daytime dysfunction.

#### Sleep quality

Sleep quality was measured using the Japanese version of the Pittsburgh Sleep Quality Index (PSQI-J) (Doi et al., 2000). The PSQI-J scale contains seven components: sleep quality (C1), sleep latency (C2), sleep duration (C3), habitual sleep efficiency (C4), sleep disturbances (C5), use of sleeping medication (C6), and daytime dysfunction (C7). Each component ranges from 0 to 3 points, and the global PSQI-J score ranges from 0 to 21, with higher scores indicating poor sleep quality. The cut-off point was 5.5, with higher scores indicating sleep disturbance.

#### Loneliness

We used a shorter 6 item version of the Drake, Loneliness Scale (De Jong Gierveld & Van Tilburg, 2006) to assess loneliness. This scale has two subscales: social and emotional loneliness. The social loneliness scale stems from the absence of a wider social network (e.g. friends, colleagues, and people in the neighbourhood; α = .84), and the emotional loneliness scale stems from the absence of an intimate relationship (e.g. a partner or best friend; α = .53). Each of the three items had a score range of 0–3 points, with total scores ≥ 2 indicating loneliness.

#### Anxiety and depressive symptoms

The Hospital Anxiety and Depression Scale (HADS) (Matsudaira et al., 2009) was used to assess anxiety and depression symptoms. It consists of two sub-factors, anxiety (α = .85) and depression (α = .74), with seven items for each sub-factor and a score range of 0–18. The higher the score, the higher the anxiety and depression scores. The cut-off points of the HADS identified possible (≥ 8) and probable (≥ 11) cases.

#### Statistical analyses

All statistical analyses were conducted using IBM SPSS version 26. Numerical data were presented as mean, standard deviation (SD), and median (min–max). Changes in sleep habits were calculated by subtracting bedtime, waking time, and sleep duration before self-restraint from during self-restraint. A one-sample t-test was performed, with 0 indicating no change as the standard. Pearson’s correlation coefficient analysis was used to investigate the correlations among demographics, sleep quality, loneliness, anxiety, and depression. Variables correlated with anxiety or depression were entered into a multiple regression analysis. Hierarchical multiple regression analysis used the stepwise method for demographic variables, loneliness as explanatory variables, and anxiety or depression as objective variables. Furthermore, a mediation analysis was conducted to examine the mediating effects of anxiety and depression, with sleep quality as an objective variable and demographic variables and loneliness as explanatory variables. The mediation analysis was conducted using model 4 of PROCESS v4.0 for SPSS, and testing was conducted using the Bootstrap method (Bootstrap sample size set at 5000). The 95% confidence interval indicates that the population mean lies within the upper and lower range of values in this sample.

## Results

### Demographic characteristics

Table 1 presents the demographic characteristics, sleep quality, and psychological variables of the participants. All participants were workers, with an average age of 46.81 years. High sleep difficulty (PSQI ≥ 5.5) was reported by 35.7% (*n* = 183), high social loneliness (≥ 2) was reported by 82.2% (*n* = 421), and emotional loneliness (≥ 2) was reported by 37.9% people (*n* = 194). The possible and probable cases of HADS - anxiety were 25.6% (*n* = 131), and the possible and probable cases of HADS – depression were 49.0% (*n* = 251).

### Sleep habits before and during self-restraint

The differences in bedtime, waking time, and sleep duration were calculated by subtracting the time before self-restraint from the time during self-restraint. A one-sample t-test was performed, with zero as the criterion for no change. Bedtime (weekdays: Mean = −0.04, *SD* = 0.49, *t* = −1.78, *df* = 511, *p* = .076, 95% CI [−0.08–0.00]; weekends: Mean = 0.02, *SD* = 0.56, *t* = .75, *df* = 508, *p* = .455, 95% CI [−0.03–0.07]) observed no difference, but in waking time (weekday: Mean = 0.08, *SD* = 0.49, *t* = 3.81, *df* = 511, *p* < .001, 95% CI [0.04–0.13]; weekends:    0.13, *SD* = 0.78, *t* = .3.69, *df* = 508, *p* < .001, 95% CI [0.06–0.20]) we found significant difference. There was a difference in sleep duration (weekdays: Mean = 0.13, *SD* = 0.59, *t* = 4.98, *df* = 511, *p* < .001, 95% CI [0.08–0.18]; weekends: Mean = 0.09, *SD* = 0.84, *t* = 2.55, *df* = 508, *p* = .011, 95% CI [0.02–0.17]). The difference in waking time was, on average, 4 min later on weekdays and 7 min later on weekends. Correspondingly, the difference in sleeping time was, on average, 7 min later on weekdays and 5 min later on weekends.

### Association factor of sleep quality, anxiety, and depressive symptoms

As shown in Table 2, the PSQI global score was negatively associated with marital status and positively associated with chronic disease, mental disorders, sleep disorders, social loneliness, emotional loneliness, anxiety, and depression. Anxiety was negatively associated with age and marital status and positively associated with mental disorders, the PSQI global score, and social and emotional loneliness. A similar correlation was observed for depression.

**Table 2:** . Correlation coefficients for main study variables.

	1	2	3	4	5	6	7	8	9	10	11	12	13	14	15	16	17
1. Age																	
2. Gender	.11*																
3. Educational level	−.11*	.27**															
4. Marital status	.33**	0.07	0.07														
5. Employment status	−0.05	.11*	0.08	−0.01													
6. Physical isolated experience	−0.05	−.10*	0.06	0.04	−0.04												
7. Children aged 0–5 years	−.20**	0.03	.13**	.24**	0.06	.11*											
8. Children aged 6–17 years	−.10*	0.01	0.01	.30**	−0.03	0.08	.22**										
9. Chronic disease	.29**	.09*	−0.06	0.08	−0.05	−0.01	−0.07	−0.01									
10. Mental disorder	−0.05	0	0.04	−0.08	−0.03	0.01	0.01	−0.04	0.05								
11. Sleep disorder	0.08	0.08	0.02	−0.06	−0.01	0.04	−0.04	−0.07	.20**	.27**							
12. Acute illness	−0.03	0	0.01	0.02	−0.05	.10*	0.02	0.07	−0.05	0.02	0.04						
13. Sleep quality (PSQI)	−0.02	−0.05	0.01	−.11*	−0.02	0.05	−0.04	−0.07	.11*	.26**	.25**	−0.02					
14. Social loneliness (DJGLS)	0.01	.13**	−0.03	−0.08	0.04	−0.07	0.02	−0.08	0.04	0.06	0.02	−0.02	.16**				
15. Emotional loneliness (DJGLS)	−.23**	−.14**	0.04	−.09*	−0.04	0.07	0.06	0	−0.02	.13**	0.02	0.02	.33**	0.06			
16. Anxiety (HAD)	−.21**	−.10*	−0.06	−.17**	−0.01	−0.01	0	−0.07	−0.01	.16**	0.05	0	.45**	.19**	.50**		
17. Depression (HAD)	−.21**	0.01	−0.03	−.18**	0.07	0.01	0.03	−0.01	−0.02	.12**	0.04	0.05	.34**	.28**	.37**	.61**	

Multiple regression analysis was used stepwise with demographic variables and loneliness as explanatory variables and anxiety or depression as objective variables (Tables 3 and 4). The main effect of anxiety was significantly associated with emotional loneliness, social loneliness, marital status, and mental disorders. Regarding depression, the main effect was significantly associated with emotional loneliness, social loneliness, marital status, employment status, and age.

**Table 3:** . Stepwise multiple regression predicting anxiety.

	B	Std. Error	Beta	t	Sig.
*F* (4,462)=466, *p* < .001					
(Constant)	2.868	0.405		7.074	*p* < .001
Predictors, (*R*^2^ = 0.286)					
Emotional loneliness (DJGLS)	1.487	0.128	0.462	11.595	*p* < .001
Social loneliness (DJGLS)	0.465	0.128	0.144	3.632	*p* < .001
Marital status	−0.708	0.268	−0.105	−2.64	*p* < .01
Mental disorder	0.961	0.457	0.084	2.106	*p* < .05

**Table 4:** . Stepwise multiple regression predicting depression.

	B	Std. Error	Beta	t	Sig.
*F* (5,461) =466, *p* <.001					
(Constant)	4.674	0.844		5.538	*p* < .001
Predictors, (*R*^2^ = 0.236)					
Emotional loneliness (DJGLS)	1.247	0.162	0.324	7.707	*p* < .001
Social loneliness (DJGLS)	0.945	0.159	0.244	5.955	*p* < .001
Marital status	−0.909	0.352	−0.112	−2.583	*p* < .01
Employment status	−0.883	0.397	−0.091	2.226	*p* < .05
Age	−0.028	0.013	−0.092	−2.076	*p* < .05

### Analysis of the mediating effects of anxiety or depression

**Figure 1.**
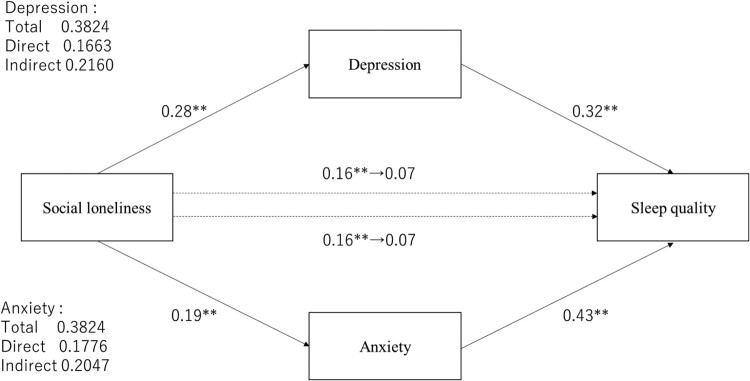
The mediating effect of depression or anxiety on social loneliness and PSQI global score. *p < .05, **p < .01.

**Figure 2.**
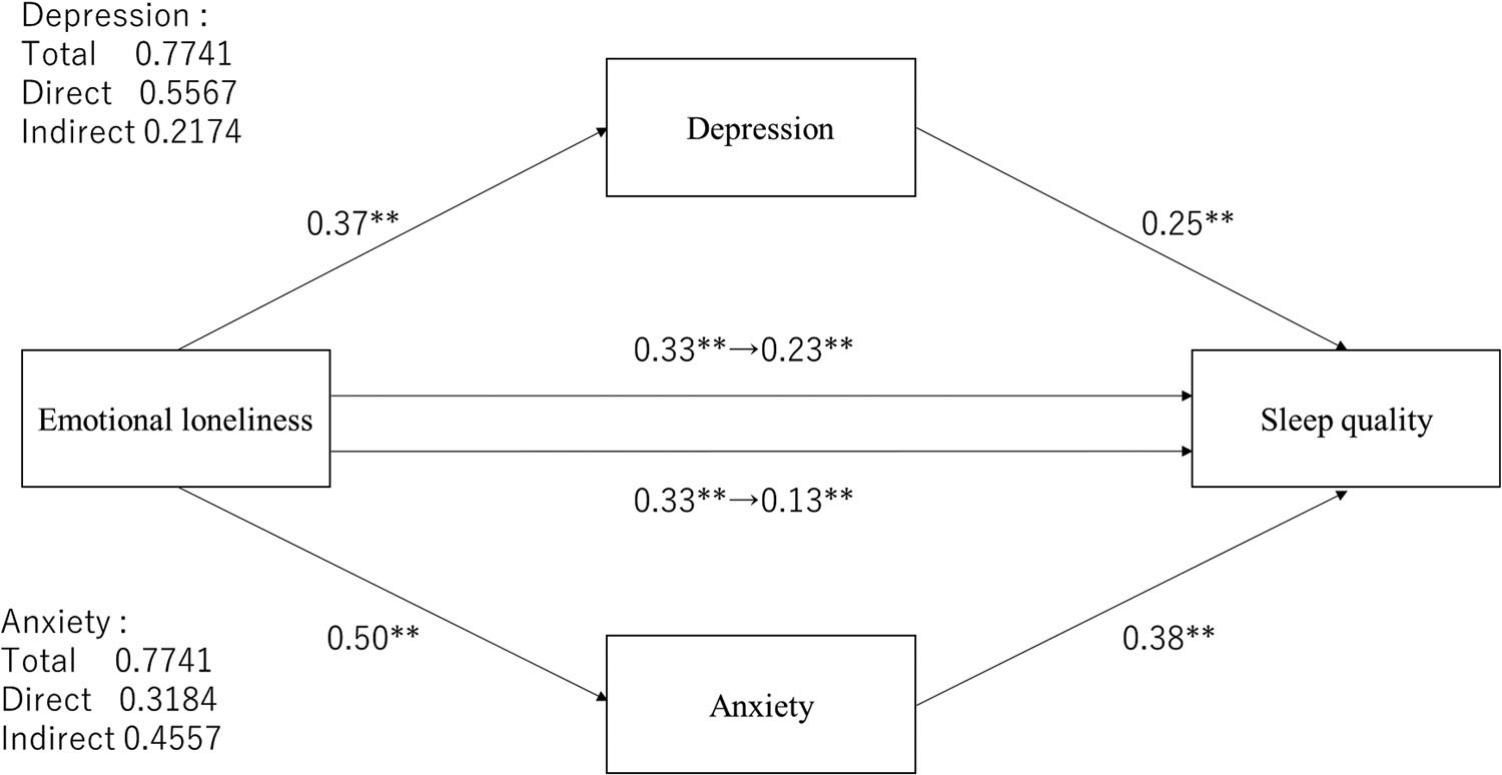
The mediating effect of depression or anxiety on emotional loneliness and PSQI global score. *p < .05, **p < .01.

We used mediation analyses to test whether demographic variables and loneliness mediated anxiety or depression to affect sleep quality. Mediated anxiety changed the effect of social loneliness on sleep quality from 0.16 (*p* < .001) to 0.07 (*p* = .069) compared to unmediated anxiety (Figure 1). The indirect effect was 0.20. To test whether this value was higher than 0, we evaluated a 95% confidence interval with the bootstrap method (number of resampling was 5000). The results indicated a significant indirect effect that did not include 0 (0.12–0.30). The mediated depression changed the direct effect of social loneliness on sleep quality from 0.16 (*p* < .001) to 0.07 (*p* = .069), and indirect effect was 0.22 (95% CI [0.13–0.31]). Mediated anxiety or depression changed the direct effect of emotional loneliness on sleep quality from 0.33 (*p* < .001) to 0.13 (*p* = 0.003) and from 0.33 (*p* < .001) to 0.23 (*p* < .001), respectively (Figure 2). Indirect effect in anxiety was 0.46 (95% CI [0.33–0.61]) and in depression, it was 0.22 (95% CI [0.13–0.31]); both mediated effects were significant.

**Figure 3.**
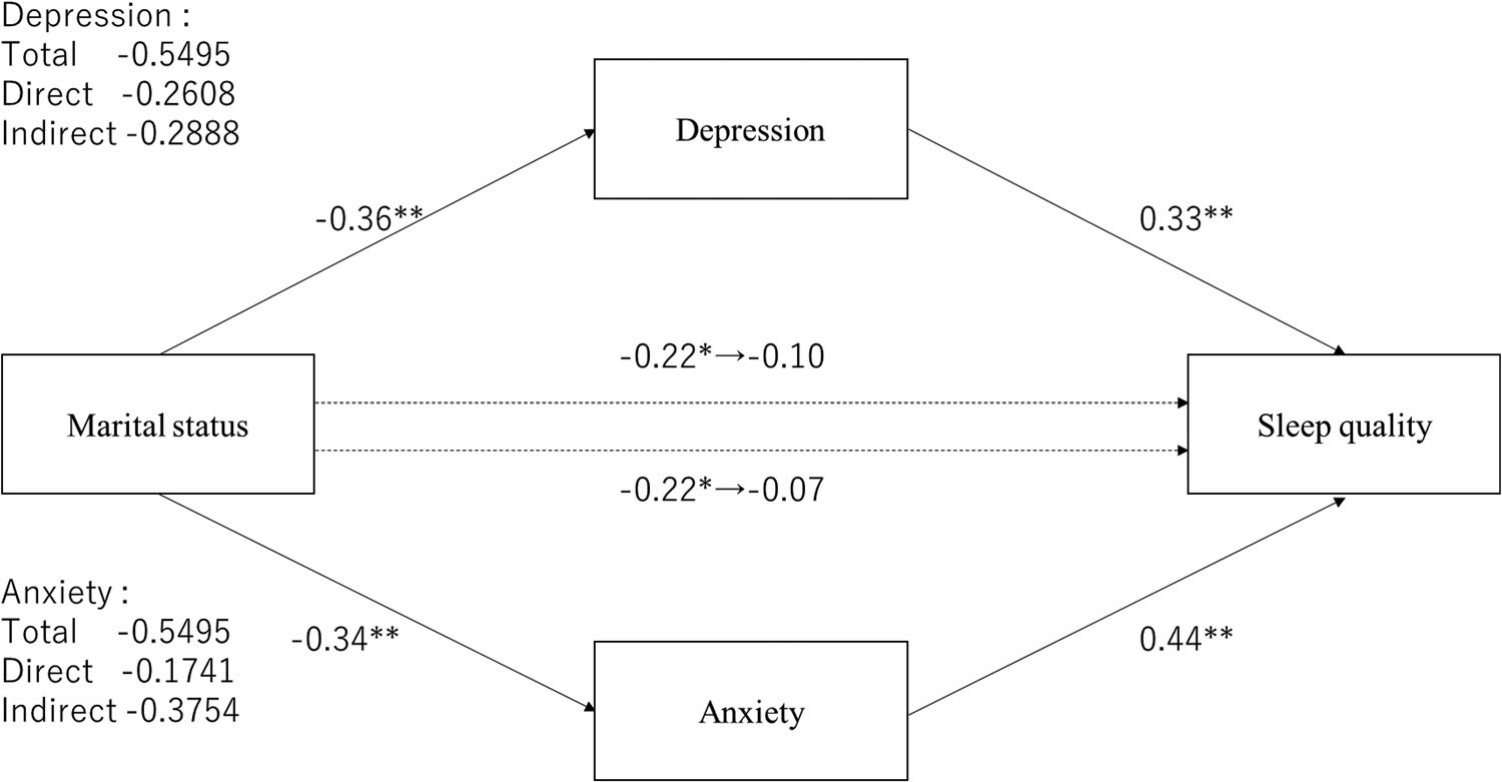
The mediating effect of depression or anxiety on marital status and PSQI global score. *p < .05, **p < .01.

**Figure 4.**
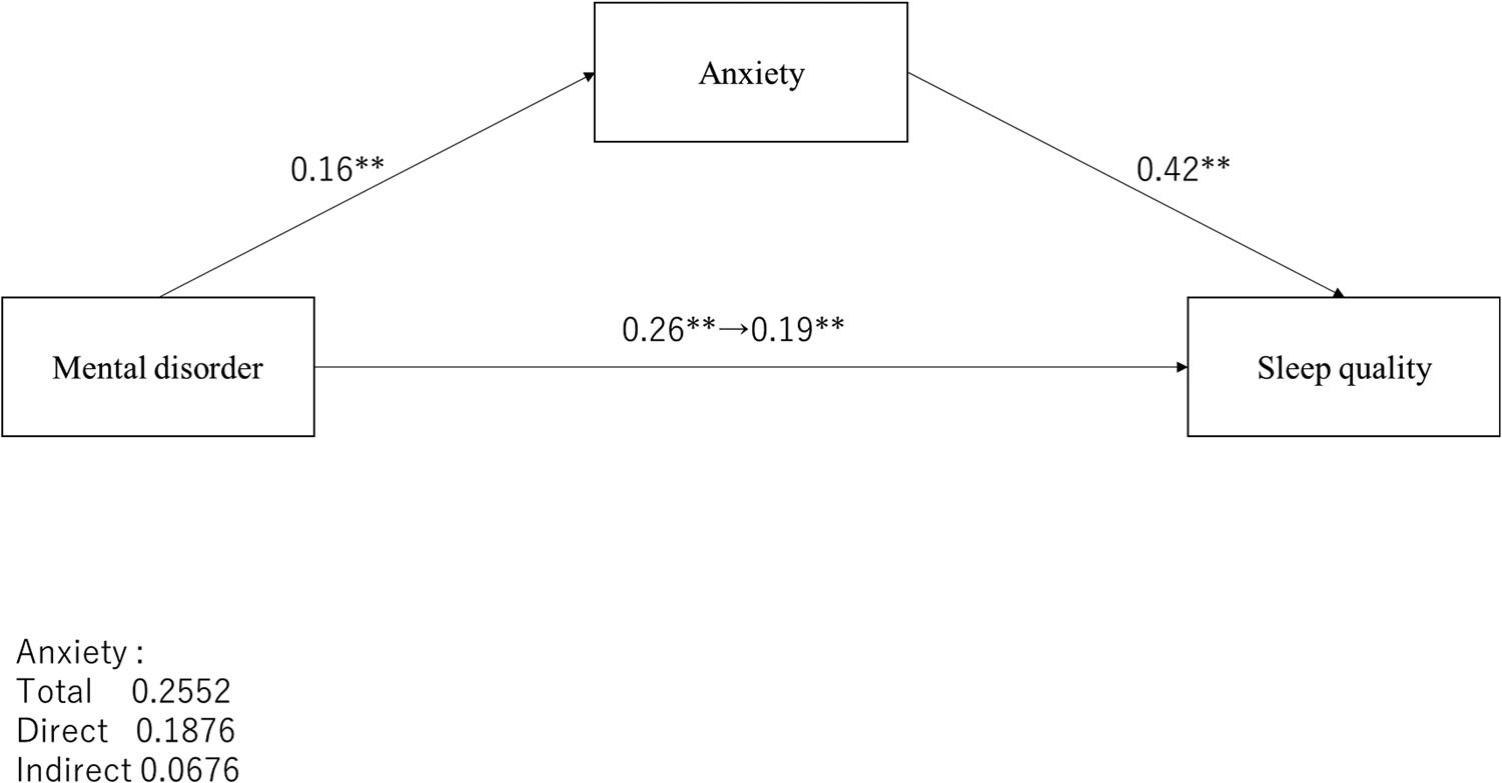
The mediating effect of anxiety on mental disorder and PSQI global score. *p < .05, **p < .01.

The direct effect of marital status on sleep quality ranged from −0.22 (*p* = .013) to −0.07 (*p* = .389) and from −0.22 (*p* = .013) to −0.10 (*p* = .222), respectively (Figure 3); anxiety or depression mediated effects were not significant. The indirect effect on anxiety was −0.38 (95% CI [−0.60–−0.17]) and on depression was −0.29 (95% CI [−0.46–−0.14]); both mediated effects were significant. Mental disorders showed only a significant relationship with anxiety (Figure 4), and the direct effect of mental disorders was significantly changed from 0.26 (*p* < .001) to 0.19 (*p* < .001). Furthermore, the indirect effect of anxiety was 0.07 ([0.03–0.11]), which was significant. Employment status and age, which were significantly associated with depression in the multiple regression analysis (Table 4), did not show a significant association in mediation analyses.

## Discussion

Although the declaration of a state of emergency in Japan included a milder behaviour regulation as compared to other countries, the present study showed increased loneliness and a low mental state. The international comparison of loneliness using the DJGLS short version before the COVID-19 pandemic indicated that the scores placed Japan in a middle position between Western and Eastern European countries (De Jong Gierveld & Van Tilburg, 2010). Conversely, we showed that these scores were much higher than those of Eastern European countries. In particular, social loneliness due to a lack of social networking increases during self-restraint, similarly, emotional loneliness due to the absence of an intimate relationship, increases as well. Probable causes of anxiety and depression in the HADS were approximately 25% in outpatients before the pandemic (Matsudaira et al., 2009). Our data showed anxiety was at 25.7% and depression was at 48.9%, which comes close to the outpatients’ mental state. A study conducted 10 days after the declaration of a state of emergency (Wakashima et al., 2020) showed similar results, confirming the psychological impacts of the mild behavioural restrictions. One reason for this could be the major changes in the working environment of Japanese workers. Despite the mild self-restraint, the number of subway passengers during commuting hours in this study period fell by up to 68% (Karako, Song, Chen, Tang, & Kokudo, 2021), thus indicating that many people did not go out. In addition, only 16.6% of Japanese workers telecommuted in November 2019 (Ministry of Land, Infrastructure, Transport and Tourism, 2020). Therefore, the sudden switch to telecommuting was challenging because IT, employment, and wage systems to support telecommuting have not been developed (Okubo, 2020). Furthermore, Japanese corporate culture values face-to-face communication (Okubo, 2020), and being unable to meet people in person may be a contributing factor to stress.

Sleep habits remained almost regular, with late waking time and longer sleep duration. Sleep-wake habits before self-restraint were based on the participants’ self-reported accounts of past rhythms and could not be assessed at that time. This may have been affected by recall bias, thus, it is conceivable that the difference between past and present sleep habits was reported as being smaller than the actual amount. However, the sleep quality during self-restraint may have improved. A large-scale Japanese population survey conducted before the COVID-19 pandemic showed that the PSQI global score (Nomura, Inoue, Kusumi, Oka, & Nakashima, 2008) and sleep difficulty (PSQI ≥ 5.5) (Aritake et al., 2015) were higher than those in this study. Our data showed fewer poor sleepers in comparison to previous studies. In Gualano et al.’s (2020) study, the risk factors of sleep disturbance during the COVID-19 pandemic implicated an abundance of women and people with chronic conditions. Healthcare workers indicated having poorer sleep quality and stronger influence of these risk factors on sleep quality (Sahin, Aker, Shain, & Karabekioğlu, 2020; Yılmaz, Kıraç, & Sahin, 2021). However, our participants included fewer women and people with chronic illnesses, which might explain the low sleep difficulty observed in this study. Additionally, because all the participants had to work, including online modalities, they had somewhat regular sleep-wake habits. This result might be because self-restraint did not significantly affect sleep quality; instead, it reduced commuting time and allowed more time for sleep.

Common factors of anxiety and depressive symptoms were social and emotional loneliness and marital status. The current results are consistent with those of several previous studies indicating that loneliness affected anxiety or depressive symptoms during the COVID-19 pandemic in the general adult population (Pai & Vella, 2021). In addition, age and marital status were associated with anxiety and depression, suggesting the importance of having connection with other people.

The global social network size increased until young adulthood and then decreased (Wrzus, Hänel, Wagner, & Neyer, 2013); thus, it is possible that there was no major change in older people during the pandemic. Conversely, young adults have larger personal or friendship networks (Wrzus et al., 2013), and the greater gap before and during behavioural restriction might be related to depressive symptoms. Being married suggests that the person has a valuable person to communicate with face-to-face, which may affect their mental health. According to the Information and Communications White Paper in Japan (Ministry of Internal Affairs and Communications, 2021), individual Internet usage rate in Japan was 89.8% in 2019 and remained almost unchanged at 83.4% in 2020; therefore, it is unlikely that social media use changed dramatically during the COVID-19 pandemic. The service most often used during the first declaration of emergency was Internet shopping. Daily social networking use in 2020 was 48.6% of everyday use, less than the 73.4% for online shopping. This is rather less than the 69.0% of social networking use in 2019. These data show that changes in daily life took precedence over communication during COVID-19 pandemic. These findings suggest that maintaining social relationships to avoid feeling lonely is crucial for maintaining mental health under physical isolation.

Emotional loneliness has a direct negative effect on sleep quality even without mental health mediation. On the other hand, it did not show a direct effect of social loneliness or marital status, but only a mediating effect on mental health. This does not mean that high social loneliness or not being married (or cohabiting) is, in itself, a negative factor, but that it may act as a negative factor for sleep when mental health deteriorates. In particular, for high emotional loneliness, it is necessary to attend to its direct effects on poor sleep quality. A recent meta-analysis confirmed that loneliness correlates with stress-detectable biological and medical factors, suggesting that measuring psychological loneliness facilitates early detection of chronic stress (Campagne, 2019).

The results of this study indicated that emotional loneliness may also predict sleep quality. To maintain good sleep quality under behavioural regulations, it may be necessary to intervene in the maintenance of regular sleep-wake rhythms and psychological factors such as loneliness, anxiety, and depression. Self-restraint in Japan during the COVID-19 pandemic was based on a specific culture but could be applied to other contexts. The present results are similar to behavioural restrictions that isolate people, such as the elderly and ill individuals, and suggest intervention strategy to maintain sleep and mental health.

This study had several limitations. First, it used a cross-sectional design; sleep-wake habits before the behavioural restriction were based on the participants’ self-reported accounts of past rhythms, and thus, could not be assessed at that time. Longitudinal studies on changes or habituation with repeated requirements of behavioural restriction are needed. Second, the participants were limited to workers; however, this sample of 512 participants was small. While the number of participants was sufficient for the purpose of the present study, which was to identify overall trends among Japanese workers, it was insufficient for detailed comparisons by type of occupation, remote work situation, or commuting time. Third, all of these results were assessed by self-report, and it may be necessary to use objective measures such as actigraphy for sleep quality and sleep-wake rhythms.

### Conclusions

To our knowledge, a model of mental health affecting the sleep quality of workers during the first self-restraint in Japan has never been reported before. The main findings of this study showed that psychological factors affect subjective sleep quality. Our results revealed that social or emotional loneliness increases anxiety or depression, resulting in anxiety-or depression-mediated loneliness and poorer subjective sleep quality.

## Data Availability

The data that support the findings of this study are available from the corresponding author upon reasonable request.
